# Attenuation of *Pseudomonas aeruginosa* Quorum Sensing Virulence of Biofilm and Pyocyanin by mBTL-Loaded Calcium Alginate Nanoparticles

**DOI:** 10.3390/polym14173655

**Published:** 2022-09-02

**Authors:** Esra Kamal Eltayb, Fulwah Yahya Alqahtani, Hamad M. Alkahtani, Ibrahim A. Alsarra, Rihaf Alfaraj, Fadilah Sfouq Aleanizy

**Affiliations:** 1 Department of Pharmaceutics, College of Pharmacy, King Saud University, Riyadh 11495, Saudi Arabia; 2 Department of Pharmaceutical Chemistry, College of Pharmacy, King Saud University, Riyadh 45142, Saudi Arabia

**Keywords:** *Pseudomonas aeruginosa*, quorum sensing, calcium alginate, nanoparticles, biofilm, pyocyanin, adhesion, A549 cells, LL 29 cells

## Abstract

*Pseudomonas aeruginosa* contributes to many chronic infections and has been found to be resistant to multiple antibiotics. Pseudomonas use a quorum sensing system (QS) to control biofilm establishment and virulence factors, and, thus, quorum sensing inhibitors (QSIs), such as meta-bromo-thiolactone (mBTL), are promising anti-infective agents. Accordingly, this study intended to investigate the antibacterial and anti-virulence activity of mBTL-loaded calcium alginate nanoparticles (CANPs) against *Pseudomonas aeruginosa* and different QS mutants. The results show that the mBTL-CANPs had higher antibacterial activity, which was made evident by decreases in all tested strains except the ∆lasR/∆rhlR double mutant, with MIC_50_ (0.5 mg/mL) of mBTL-CANPs compared with free mBTL at MIC_50_ (˃1 mg/mL). The biofilm formation of *P. aeruginosa* and some QS-deficient mutants were reduced in response to 0.5–0.125 mg/mL of mBTL-encapsulating CANPs. The pyocyanin production of the tested strains except ∆lasA and ∆rhlR decreased when challenged with 0.5 mg/mL of mBTL-loaded NPs. The subsequent characterization of the cytotoxic effect of these NPs on human lung epithelial cells (A549) and cystic fibrosis fibroblast cells (LL 29) demonstrated that synthesized NPs were cytocompatible at MIC_50_ in both cell lines and markedly reduced the cytotoxic effect observed with mBTL alone on these cells. The resulting formulation reduced the *P. aeruginosa* strains’ adhesion to A549 comparably with mBTL, suggesting their potential anti-adhesive effect. Given the virulence suppressing action, cytocompatibility, and enhanced anti-biofilm effect of mBTL-CANPs, and the advantage of alginate-based NPs as an antimicrobial delivery system these nanoparticles have great potential in the prophylaxis and treatment of infection caused by *Pseudomonas aeruginosa*.

## 1. Introduction

Infections caused by multidrug-resistant pathogens kill over 700,000 people worldwide each year, with the number expected to rise to 10 million by 2050 [1]. This is due to the abuse and overuse of antibiotics leading to the emergence of multidrug-resistant (MDR) bacteria [2]. *Pseudomonas aeruginosa* is a Gram-negative bacterium that causes pneumonia, infection of the bloodstream, urinary tract, burn wounds, and surgical sites and plays a crucial role in cystic fibrosis. Previous reports have found that *P. aeruginosa* was resistant to many antibiotics [3,4], and, thus, *P. aeruginosa* is positioned at the top of the World Health Organization’s priority pathogen list (critical) for novel antibiotic development against MDR pathogens [5]. Therefore, alternative therapeutic approaches are urgently needed to address the growing threat of multi-antibiotic resistance among *P. aeruginosa* strains [6,7,8].

Quorum sensing (QS) is a cell–cell communication mechanism that regulates *P. aeruginosa* virulence factor production involving biofilm development and pyocyanin production via countering signal molecules called autoinducers (AIs) [9]. There are three well-known QS pathways in *P. aeruginosa*, including two LuxI/R-type coordination (lasI/R and rhlI/R) and a Pseudomonas quinolone signal system (PQS) [10]. The outcome is that QS controls much of the virulence of *P. aeruginosa*, and numerous other infections have paved the way for a novel therapeutic target [11,12,13]. In this aspect, several studies have shown that inhibiting QS using quorum sensing inhibitors (QSIs) reduces virulence factors and biofilm formation [13,14,15]. Therefore, interfering with QS is believed to be a promising alternative tactic to combat multidrug-resistant *P. aeruginosa* [16]. In this regard, a previous study found that the synthetic compound meta-bromo-thiolactone (mBTL) inhibited the *P. aeruginosa* QS receptors *lasR* and *rhlR* [13]. mBTL inhibited pyocyanin formation and increased nematode persistence in a *P. aeruginosa* infection model [13].

Alginate is a natural polysaccharide made up of b-D-mannuronic acid (M block) and a-L-guluronic acid (G block) [17,18]. Alginate is widely used in the preparation of nanoparticles (NPs) for drug delivery because it is biocompatible, non-toxic, and easily degradable [17,18]. Moreover, alginate oligosaccharides have also been shown to interrupt biofilm production and maturation, as well as to enhance antibiotic activity against *P. aeruginosa* [19]. In our study, mBTL as a QSI was successfully obtained in calcium alginate nanoparticles (CANPs) with the optimal nano-size and desired physicochemical properties [20]. Therefore, the aim of this study was to subsequently examine the antibacterial outcome of mBTL-loaded CANPs, and their potential in defeating QS-related phenotypes, including biofilm and pyocyanin formation of *P. aeruginosa* and different QS mutants. Furthermore, the cytotoxicity of these NPs on A549 and LL 29 cell lines and their ability to interrupt the adhesion of *P. aeruginosa* strains to A549 cells were also studied.

## 2. Materials and Methods

### 2.1. Bacterial Strains and Growth Conditions

The strains of *P. aeruginosa* PAO1 wild-type and mutants were obtained from the library of PAO1 transposon from the University of Washington Genome Centre (Washington University, Washington, WA, USA) [21]. The bacterial strains used in this research were described in Table 1. All isolates were cultured in Luria–Bertani broth (LB) or LB agar or cetrimide agar supplemented with tetracycline 100 µg/mL as required at 37 °C for 18 h. The culture media were purchased from Oxoid (Basingstoke, Hampshire, UK).

### 2.2. Growth Curve of Bacterial Isolates

The effects of plain and mBTL-loaded CANPs on bacterial growth were determined according to the Clinical and Laboratory Standard Institute (CLSI) protocols and as described previously [22,23]. The NPs were serially diluted in LB in standard Bioscreen C 96-well microtiter plates at twofold serial dilutions ranging from a 1 to 0.125 mg/mL concentration (Labsystems Oy, Helsinki, Finland). The bacterial suspension at the exponential phase was adjusted to 0.5 McFarland standard. The inoculated plates were incubated for 20 h at 37 °C with continuous shaking, and measurements of optical density (OD) were recorded every 1-h interval at 600 nm. To eliminate the background, the OD reading of the tested NPs at each concentration and LB medium alone were subtracted from the OD of the wells inoculated with bacteria.

### 2.3. Biofilm Formation Assay 

The effect of mBTL-loaded CANPs on *P. aeruginosa* biofilm development was investigated as formerly reported with slight modification [24]. An overnight culture of *P. aeruginosa* in LB broth was adjusted to 0.5 McFarland standard. Then, 100 µL was added to 100 µL of LB containing different concentrations of mBTL-loaded CANPs ranging from 1 mg/mL to 0.125 in a 96-well microtiter plate (Thomas science), and it was incubated at 37 °C overnight. Bacterial broth suspension was used as a positive control well, while the medium only was used as a negative control well. Planktonic cells were decanted, then the plates were washed twice with 0.9% NaCl and upturned to dry at room temperature for 1 h. Later, 150 µL of crystal violet solution (CV; Prolab Diagnostics) was added to the wells in order to dye the adherent bacteria for 15 min. Then, the excess stains in the wells were removed, and they were washed 3 times with 0.9% NaCl. The bound CV was then solubilized in 200 µL of ethanol–acetone (80:20 *v*/*v*). Finally, the absorbance of the stained adherent bacteria was measured at 595 nm using a microplate reader (BioTek Instruments, Winooski, VT, USA). 

### 2.4. Pyocyanin Production Assay 

The effect of mBTL-loaded CANPs against *P. aeruginosa* pyocyanin production was investigated. The supernatant of the *P. aeruginosa* strains that were cultured overnight in the presence of mBTL-loaded CANPs was collected and mixed with chloroform. The chloroform layer containing pyocyanin was collected and extracted with 0.2 M HCL, vortexed, and centrifuged for 2 min at 8000 rpm. The absorbance of the pink HCl layer was quantified at 520 nm using a plate reader (BioTek).

### 2.5. Cytotoxicity Studies

As previously reported [25], AlamarBlue assay was used to evaluate the cytotoxicity of CANPs. A549 cells (human lung adenocarcinoma cell line CCL 185, ATCC) and LL 29 (lung fibroblast CCL-134, ATCC) were seeded in 24-well plates in DMEM or RPMI-1640 supplemented with 10% FBS and 1% antibiotic–antimycotic solution, respectively, overnight, until reaching 70% to 90% confluency. Then the cells were treated with CANPs (1–0.0156 mg/mL) for 24 and 48 h. Subsequently, the cells were seeded with fresh DMEM or RPMI-1640 media containing 10% AlamarBlue solution, and the cells were incubated for 2 to 4 h at 37 °C and 5% CO2. The fluorescence was measured using SpectraMax M5 fluorometer (Molecular Devices, CA, USA) at 550 nm excitation and 590 nm emission wavelengths. After eliminating the signal from the media alone from both samples, the viability of the treated cells was expressed as a ratio to the non-treated cells as follows:

Cell Viability (%) = (Fluorescence of cells treated with CNPs)/(Fluorescence of non-treated cells) × 100

### 2.6. Bacterial Adhesion Assay 

The adhesion analysis was performed using A549 cells as described in a previous study with minor modifications [26]. A549 cells were cultured in 24 well plates at ~7.5 × 10^4^ cells/well and incubated until they reached a confluency of about 85%. Then, the medium was replaced with serum-free medium for 2 h. After that, the medium was changed to medium containing 0.5 mg/mL of mBTL and mBTL-loaded CANPs, respectively. *P. aeruginosa* was added to the A549 cells at a multiplicity of infection (MOI) of 100 (bacteria per eukaryotic cell) and incubated for 2 h at 37 °C and 5% of CO_2_. After incubation, the cells were washed three times with PBS before being treated with 0.1% saponin for 5 min, followed by serial dilution and spread on LB plates and incubated overnight at 37 °C and 5% CO_2_. Then, the numbers of adhered bacteria (colony forming units) were counted the next day.

### 2.7. Statistical Analysis

The data are presented as the mean ± standard deviation (SD). Student’s *t*-test and ANOVA tests were used when appropriate. A *p*-value of <0.05 is considered significant.

## 3. Results

### 3.1. Growth Pattern of P. aeruginosa QS Mutants Challenged with Free mBTL and mBTL-Loaded CANPs

**Figure 1:**
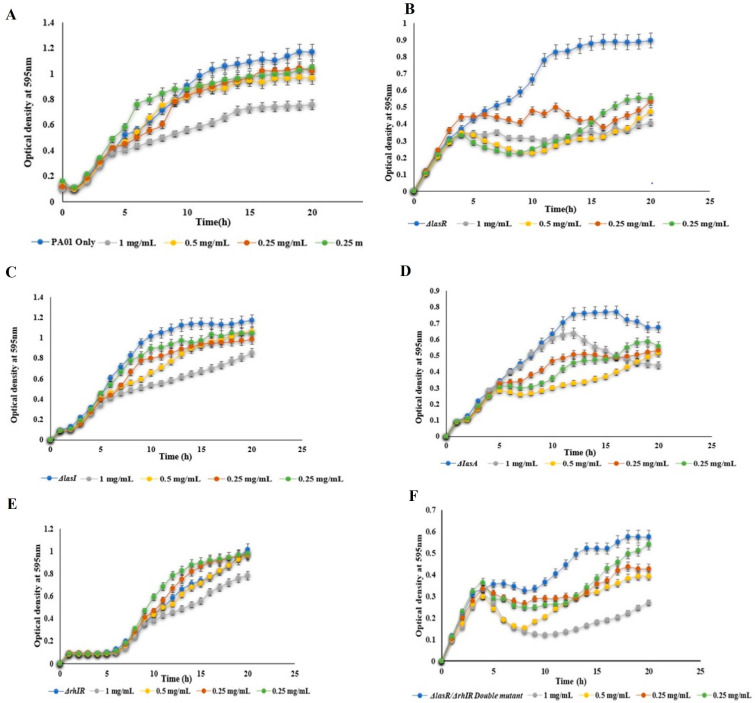
The effect of free mBTL at different concentrations was investigated using (**A**) wild type PA01 and different pseudomonal QS mutants (**B**) PA1430 (*ΔLasR*), (**C**) PA1432 (*ΔlasI*), (**D**) PA1871 (*ΔIasA*), (**E**) PA3477 (*ΔrhlR*), and (**F**) PA1871 (*ΔlasR/ΔrhlR*) double mutants. All experiment performed three times in triplicate.

**Figure 2:**
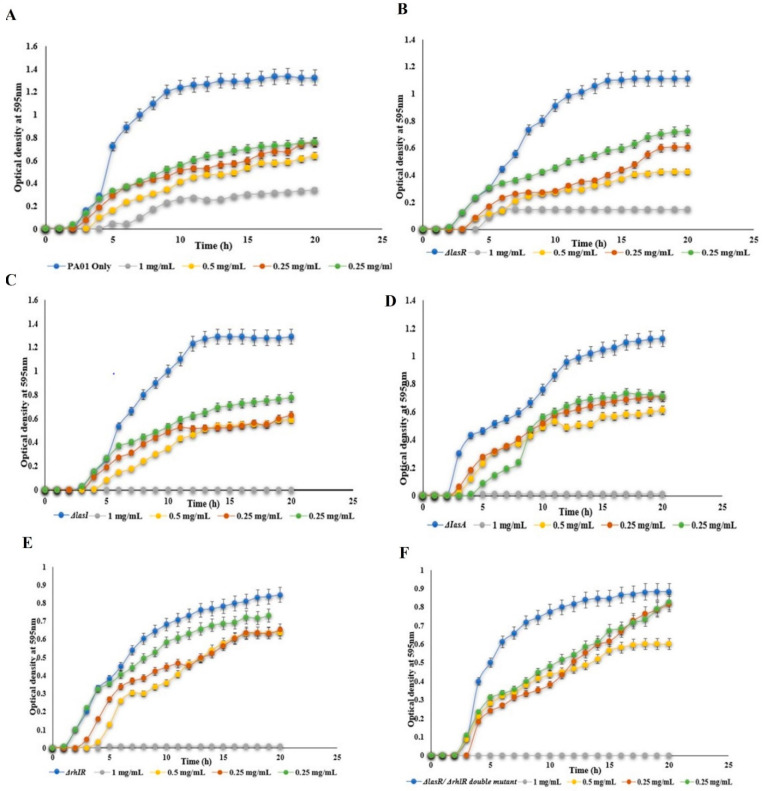
The effect of mBTL- loaded at different concentrations was investigated using (**A**) wild type PA01 and different pseudomonal QS mutants (**B**) PA1430 (*ΔlasR*), (**C**) PA1432 (*ΔlasI*), (**D**) PA3477 (*ΔrhlR*), (**E**) PA1871 (*ΔlasA)*, and (**F**) (*ΔlasR)/ΔrhlR* double mutants. All experiment performed three times in triplicate.

In our previous study [20], mBTL-loaded CANPs were prepared successfully with a particle size of 175.4 ± 7.25 nm, a zeta potential of 45.7 mv ± 0.920, and an encapsulation efficiency % of 93.21% ± 0.045. A sustained release of mTBL from the CANPs was observed. Fourier transform infrared spectroscopy (FTIR) showed a stable structure of the loaded mBTL, and differential scanning calorimetry (DSC) exhibited no interaction between the mBTL and the polymer [20]. Therefore, in the current study, the antimicrobial activity of the free mBTL and mBTL-loaded CANPs against *P. aeruginosa* WT and various QS phenotypic mutants were investigated at concentrations from 1 mg/mL to 0.125 mg/mL. The results of the screening antimicrobial effect determined by the MIC_50_ were obtained by the broth dilution method. The analysis of the bacterial growth showed a dose-dependent killing of the mBTL (Figure 1). The mBTL at 1 mg/mL reduced the bacterial growth of PA01, ΔLasR, ΔLasA, ΔRhlR, and ΔlasR/ΔRhlR double mutants after 20 h by 35.40%, 54.52%, 26.94%, 35.06%, 22.82%, and 53.08%, respectively. Meanwhile, at 1 mg/mL of mBTL-loaded CANPs and after 20 h, further reductions in bacterial killing were observed (Figure 2) with 74.28%, 86.70%, 100%, 100%, 100%, and 100% decreases in the growth of PA01, ΔLasR, ΔlasA, ΔRhlR, and ΔlasR/RhlR double mutants, respectively. At 0.5 mg/mL of mBTL-loaded CANPs, the growth of PA01, ΔLasR, ΔLasA, ΔRhlR, and ΔlasR/RhlR double mutant strains after 20 h decreased by 51.71%, 62.01%, 55.10%, 45.54%, 24.90%, and 33.06% of respectively. Of note, the same pattern of reduction in bacterial growth was observed with 0.5 mg/mL of mBTL loaded in CANPs when compared to mBTL alone. 

### 3.2. Effect of mBTL and mBTL-Loaded CANPs on Bacterial Biofilm Formation

**Figure 3:**
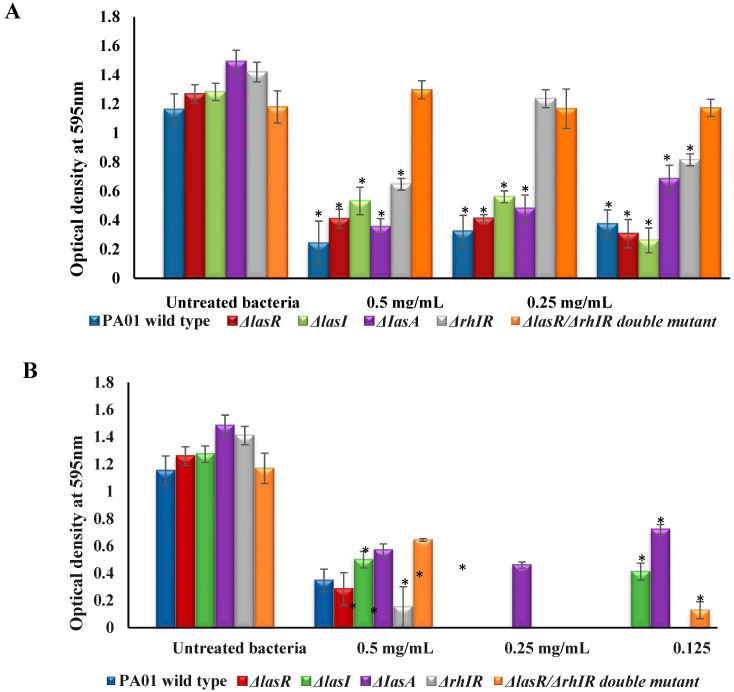
Biofilm quantification assay of *P. aeruginosa* strains. Quantifications of biofilm of PA01, *ΔlasR*, *ΔlasI*, *ΔIasA*, *ΔrhIR*, and *ΔlasR*/*ΔrhIR* double mutant were performed in the presence of 0.125–0.5 mg/mL of (**A**) mBTL or (**B**) mBTL-loaded CANPs by crystal violet assay. Experiment performed in triplicate (mean ± SD). Based on Student t test, (* *p* < 0.05) indicates significant reduction in biofilm formation when compared to untreated bacteria.

The *P. aeruginosa* strains were treated with a range of 0.125–0.5 mg/mL mBTL and mBTL-loaded CANPs to evaluate their ability to reduce or inhibit bacterial biofilms. A previous experiment showed MIC_50_ was equal to 0.5 mg/mL, so a sub-MIC concentration was also used to test the effect on biofilm production maintaining the cell viability of the tested strains. As shown in Figure 3, at MIC_50_ (0.5 mg/mL), the mBTL significantly reduced the biofilm formation (*p* < 0.05) of PA01, ΔlasR, ΔlasI, ΔIasA, and ΔrhIR by 79.16%, 67.69%, 58.53%, 76.16%, and 54.39%, respectively. The same concentration of mBTL loaded in CANPs was able to decrease the biofilm formation of PA01, ΔlasR, ΔlasI, ΔIasA, and ΔrhIR by 69.92%, 76.79%, 60.84%, 61.56%, and 89.22%, respectively. Conversely to mBTL alone, loaded CANPs reduced the biofilm formation of a double mutant (ΔlasR/ΔrhIR) by 44.92%. Remarkably, to overcome the effect of mBTL on cell viability and detect the effect only on biofilm, lower concentrations (0.125 and 0.25 mg/mL) were also studied, showing also significant reductions (*p* < 0.05) in the biofilm formation (Figure 3A,B).

### 3.3. Effect of mBTL and mBTL-Loaded CANPs on Bacterial Pyocyanin Production

**Figure 4:**
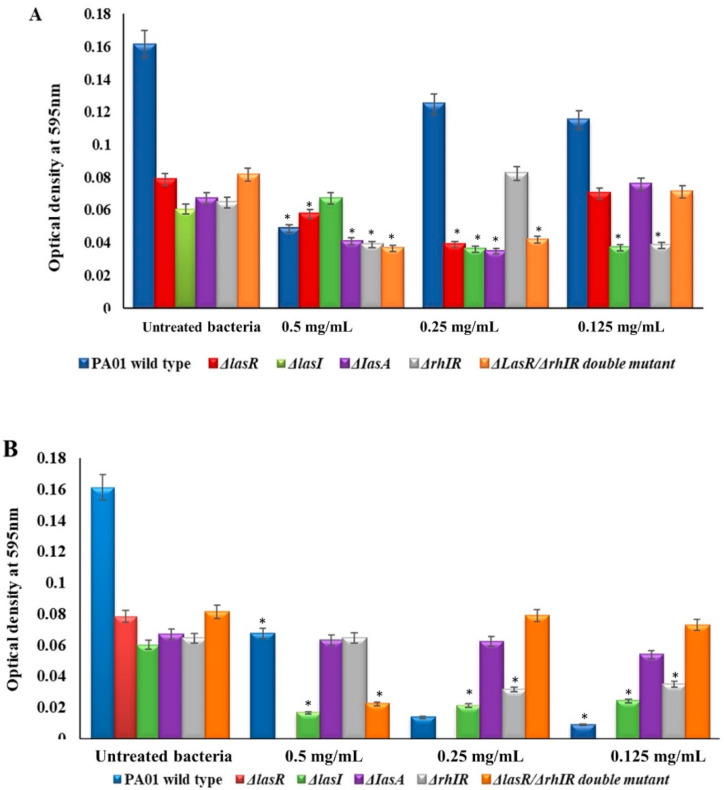
Pyocyanin production of *P. aeruginosa* strains. Levels of pyocyanin production of PA01, *ΔlasR*, *ΔlasI*, *ΔIasA*, *ΔrhIR,* and *ΔlasR*/*ΔrhIR* double mutant in the presence of 0.5–0.125 mg/mL of (**A**) mBTL or (**B**) mBTL-loaded CANPs were measured. Experiment performed in triplicate (mean ± SD). Based on Student *t*-test, (* *p* < 0.05) indicates significant reduction in pyocyanin production when compared to untreated bacteria.

In this study, the *P. aeruginosa* strains challenged with a 0.5 mg/mL concentration of mBTL alone and mBTL-loaded CANPs and the extent of pyocyanin in the supernatant were quantified. Of note, the level of pyocyanin production was high in the wild-type and limited in other mutants, as expected. Upon treatment with 0.5 mg/mL of mBTL, the results show that mBTL significantly (*p* < 0.05) inhibited the pyocyanin production of PA01 by 70.48%. To a lesser extent, mBTL decreased pyocyanin levels by 54.14%, 60.84%, 41.04%, and 45.77% in ΔlasR, ΔlasI, ΔIasA, and ΔrhIR respectively (Figure 4). The mBTL had no significant effect on the pyocyanin production of a ΔrhIR null mutant and a ΔlasR/ΔrhIR double mutant. The treatment of bacterial strains with 0.5 mg/mL of mBTL-loaded CANPs decreased the pyocyanin production of PA01 and ΔlasI by 58.20% and 72.45%, respectively. In a lasR null mutant and a ΔlasR/ΔrhIR double mutant, the loaded NPs inhibited the pyocyanin levels by 100% and 72.59%, respectively, more than mBTL alone. There was no impact of mBTL-containing NPs on the production of pyocyanin in ΔIasA and ΔrhIR mutants. Lower concentrations (0.125 and 0.25 mg/mL) were studied to overcome the cell-killing effect of the MIC_50_. The results show consistent and significant (*p* < 0.05) inhibition of pyocyanin production for only the ΔlasR strain compared to the control cells.

### 3.4. Cytotoxicity of Formulated CANPs

**Figure 5:**
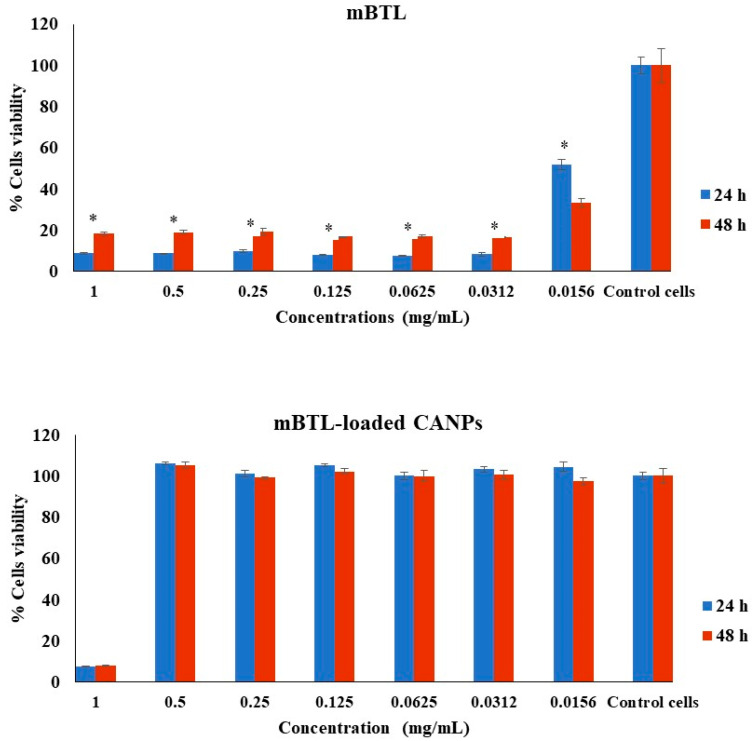
Effect of mBTL-loaded CANPs on the viability of A549 cells. The effects of mBTL and mBTL-loaded CANPs on cell viability were assessed after 24 h and 48 h. The untreated cells (control cells) were normalized to 100% viable, and the treated cells were the percentages of viable cells (treated cells) compared to the control. The data are presented as the mean ± SD of triplicate experiments. * *p* < 0.05 in Student’s *t*-test relative to the untreated control cells.

**Figure 6:**
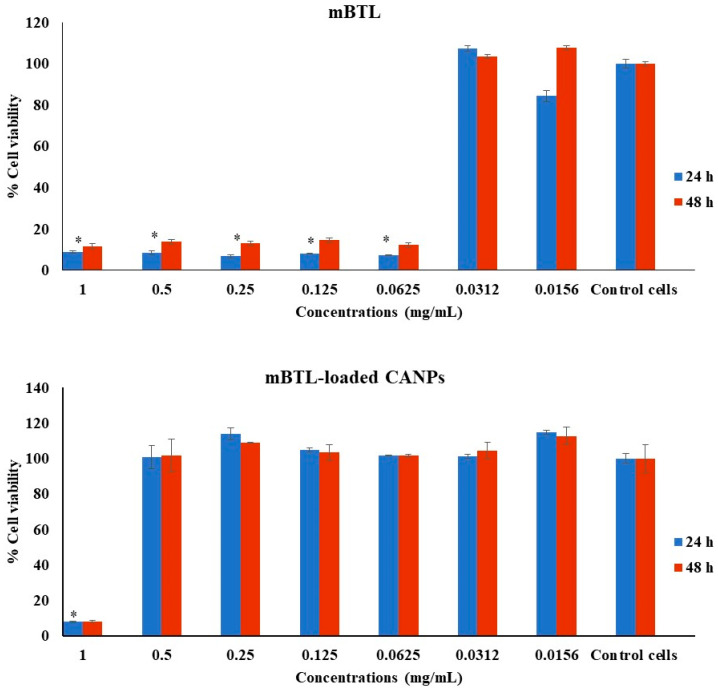
Effect of mBTL-loaded CANPs on the viability of LL 29 cells. The effects of mBTL and mBTL-loaded CANPs on cell viability were assessed after 24 h and 48 h. The untreated cells (control cells) were normalized to 100% viable, and the treated cells were the percentages of viable cells (treated cells) compared to the control. The data are presented as the mean ± SD of triplicate experiments. * *p* < 0.05 in Student’s *t*-test relative to the untreated control cells.

The results of the Alamar Blue assay revealed that exposing A549 to concentrations of mBTL of up to 0.0156 mg/mL for 24 and 48 h had significant cytotoxic effects (Figure 5). In LL 29 cells, mBTL significantly reduced the cell viability at lower concentrations of up to 0.0312 mg/mL after 24 and 48 h (Figure 6). However, the mBTL-loaded CANPs inhibited the cell viability of both A549 and LL 29 significantly only at a higher concentration (1 mg/mL) (Figure 5 and Figure 6).

### 3.5. Effect of mBTL-Loaded CANPs on the Adhesion of P. aeruginosa to Human Lung A549 Cell Line 

**Figure 7:**
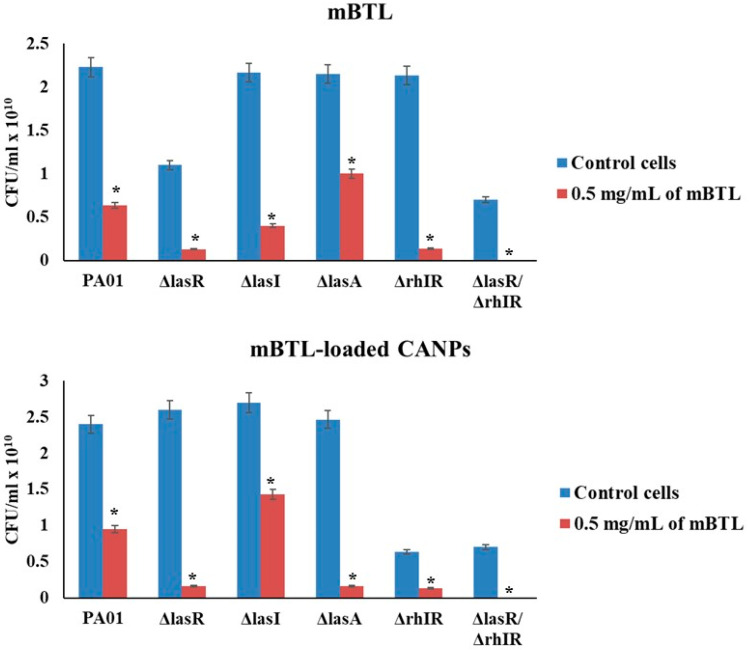
Effects of mBTL-loaded CANPs on the adhesion of *P. aeruginosa* isolates to A549 cells. Quantitative analysis of bacterial adhesion to A549 cells. Blue histogram: A549 cells infected with PA01, (*ΔlasR*), (*ΔlasI*), (*ΔIasA*), (*ΔrhIR*), and (*ΔlasR*/*ΔrhIR*) as control cells. Red histogram: A549 cells infected with wild-type and QS mutant strains and simultaneously treated with 0.5 mg/mL of mBTL or mBTL-loaded CANPs. The data are presented as the mean ± SD of triplicate experiments. * Based on ANOVA, *p* < 0.05 relative to control cells.

The adhesion of *P. aeruginosa* wild-type and different QS mutants to the surface of A549 lung epithelial cells was evaluated using the plate count method (Figure 7). Adhesion assay concentrations of 0.5 mg/mL of mBTL and mBTL-loaded CANPs were considered. The results show that mBTL significantly reduced the adhesion of PA01, PA1430 (ΔlasR), PA1432 (ΔlasI), PA1871 (ΔIasA), PA3477 (ΔrhIR), and the double mutant by 71.6%, 88.1%, 81.5%, 53.4%, 93.7%, and 100%, respectively (Figure 7). Comparably, the bacteria significantly reduced the adherence capabilities of the wild-type, PA1430 (ΔlasR), PA1432 (ΔlasI), PA1871 (ΔIasA), PA3477 (ΔrhIR), and the double mutant by 60.4 %, 93.8%, 47%, 93.2%, 78.9%, and 100%, respectively (Figure 7). Of note, the treatments with mBTL and mBTL-loaded CANPs were able to prevent the adhesion of the double mutant (Figure 7).

## 4. Discussion 

Based on a previous successful formulation of mBTL-loaded CANPs in our lab [20], these NPs were subsequently subjected to microbiological analysis and compared to free mBTL. *Pseudomonas aeruginosa* PA0 wild-type and different QS mutants, including lasR (PA1430), lasI (PA1432), IasA (PA1871), rhIR (PA3477), and a lasR and rhIR double mutant (DA6) were used. The growth of the strains challenged with increasing concentrations of mBTL was measured, and the results show dose-dependent inhibition of bacterial viability. This corroborates the findings described by O’Loughlina, C.T. et al. [13], in which mBTL mediated an effect through the partial inhibition of the bacterial QS systems LasR and RhlR [13,27]. Interestingly, loading mBTL into CANPs increased the anti-pseudomonal aureginosa effect of mBTL with considerable decreases in MIC_50_ from ˃1 mg/mL to 0.5 mg/mL for wild-type isolate, lasR, lasI, and lasA. Of note, the loaded NPs increased the sensitivity of the double mutant to mBTL at a higher concentration of 1 mg/mL. This might be due to the enhanced uptake of mBTL when loaded in CANPs which require further future studies to understand the exact mechanism.

To support the findings that mBTL acts as a QSI, QS virulence-dependent phenotypes as biofilm and pyocyanin were examined using different *P. aeruginosa* strains. In agreement with a previous study [13], mBTL reduced the biofilm formation of wild-type at a 0.5 mg/mL concentration. In addition, the biofilm formations of other single mutants were subsided by mBTL. In contrast, the biofilm formation of the double mutant was not affected by mBTL, which is consistent with a previous study [13]. This further illustrates our suggestion regarding the deletion of genes of interest that mBTL target in this strain. Of note, despite that rhIR seems to be the key in vivo target of mBTL, as described by Bassler and co-workers [13], mBTL at an MIC_50_ concentration reduced the biofilm formation of a rhIR null mutant, which was unexpected. Thus, the biofilm inhibition seen by Bassler and colleagues could be due to mBTL inducing rhamnolipids, as suggested by other researchers [28]. Remarkably, CANPs containing mBTL significantly reduced the biofilm formation of PA01, ΔlasR, ΔlasI, and ΔIasA compared to mBTL alone (at an MIC_50_ concentration). Surprisingly, the effect of mBTL-loaded NPs was stronger on ΔrhIR than mBTL alone (89.22% versus 54.39%, respectively at an MIC_50_ concentration) and obtained complete inhibition at a sub-MIC_50_ concentration. Contrary to mBTL alone, which showed no inhibition, loaded CANPs inhibited biofilm development by 44.92% in the double mutant (lasR/rhIR) at MIC_50_ and obtained a more significant reduction at a sub-MIC_50_ concentration. Such an enhancement of the mBTL-loaded CANPs in the biofilm formation of ΔrhIR and the double mutant (lasR/rhIR) could have been caused by alginate NPs, as alginates were previously reported to disrupt biofilm formation and maturation [29]. However, this might seem counterintuitive, since alginate is a major component in *P. aeruginosa* biofilm formation, and therapeutic targeting alginate is a potential antibiofilm, as described previously [30]. However, alginates used in the formulation of CANPs are obtained from algae, which differ from bacterial alginate as bacterial alginate lack the G blocks (homopolymeric regions of poly-L-guluronate) [19]. These G blocks could be a contributing component in the antibiofilm effects, as reported previously [29].

The level of pyocyanin production observed in our study was high in wild-type and limited in other mutants, as expected and as reported previously by Bassler and co-workers [13]. In agreement with others [13], the mBTL at a concentration of 0.5 mg/mL (1.4 µM) inhibited the pyocyanin production of PA01 by 70.48%, which was comparable with an 83% reduction in the pyocyanin level of *P. aeruginosa* PA14 in response to mBTL with an MIC_50_ of 8 µM. However, the effect of mBTL on the pyocyanin production of PA01 observed in our study was at a lower concentration than the one reported by others [13,28]. Gratifyingly, mBTL-loaded NPs exhibited an impact similar to that of free mBTL for PA01, with a more prominent effect on the lasR null mutant and the ΔlasR/ΔrhIR double mutant at an MIC_50_ concentration. The increased inhibition of pyocyanin of the ΔlasR/ΔrhIR double mutant in response to the mBTL-loaded CANPs when compared to mBTL alone (72.59% versus 5.52%) suggests that mBTL-NPs attenuated the pyocyanin production by a mechanism other than the lasR/rhIR gene. Interestingly, this pattern was similar to that of biofilm formation, as described earlier. Conversely, the mBTL-loaded CANPs at an MIC_50_ concentration exhibited no effect on the pyocyanin levels of the ΔIasA and ΔrhIR mutants. This was expected for the rhIR mutant, given the role of this gene, as mBTL functions in vivo by inhibiting quorum sensing via rhlR, as observed by Bassler and co-workers [13]. It was also reported that while some of the mBTL effects appear via lasR, all of the mBTL effects are governed by rhIR [13]. This explains the apparent reduction in the pyocyanin levels of the lasR null mutant in response to mBTL and the complete inhibition in the mBTL-loaded NPs at MIC_50_ and sub-MIC_50_ concentrations, which seem to have been mediated mostly through rhIR. The mBTL-loaded CANPs did not affect the pyocyanin production of ΔIasA, while mBTL alone did; this may have been caused by the masking effect of NPs. 

As shown in this study, the mBTL-loaded CANPs exhibited higher antimicrobial and comparable anti-virulence activities against the tested isolates when compared to mBTL alone. These outcomes are in line with an earlier study where modified alginate NPs loaded with ciprofloxacin and QSI 3-amino-7-chloro-2-nonylquinazolin-4 (3H) -one (ACNQ) cleared the infection in a 3D ex vivo skin model [31]. Another recent study found that the anti-C. perfringens activity of EntDD14 was enhanced when loaded in alginate NPs [32]. This was also observed with other alginate-based NPs; loading nisin onto chitosan/alginate NPs increased the anti-Staphylococcus aureus action of this bacteriocin [33]. Moreover, sodium alginate/gum acacia NPs loaded with isometamidium HCl (ISMM), which is used for the treatment of trypanosomiasis, showed more enhanced trypanocidal activity than pure ISMM [34]. The ISMM released from the NPs showed a sustained pattern and minimum side effects [34].

In the present study, the cytotoxicity of mBTL and mBTL-loaded CANPs was evaluated in A549 and LL 29 cells. The mBTL alone was found to be toxic to A549 and LL 29 even at lower concentrations of up to 0.0156 and 0.0312 mg/mL, respectively. Interestingly, the mBTL-loaded CANPs did not inhibit the cell viability of A549 and LL 29 cells up to a concentration of 0.5 mg/mL. Similar to our findings, a previous study showed that empty sodium alginate/gum Arabic NPs had no inhibitory effect on different cancer cell proliferation, including A549, at different concentrations reaching 100 µg/mL [35]. These results revealed the cytocompatibility of the CANPs prepared in this study. However, at a higher concentration (1 mg/mL), both formulated NPs were toxic to the cells over 24 and 48 hr. The mechanism of this cytotoxic effect is still unclear; however, increased concentrations of NPs might induce cell apoptosis, necessitating further future studies. According to our results, the encapsulation of mBTL into CANPs significantly decreased its cytotoxicity in A549 and LL 29 cells when compared to mBTL alone, as shown by the enhanced uptake of the encapsulated mBTL by *P. aeruginosa* strains, as observed earlier. In agreement with our study, the formulation of ISMM in sodium alginate/gum acacia NPs found to reduce the cytotoxicity and hemolysis associated with the trypanocidal drug alone [34]. The cytocompatibility of mBTL-CANPs on A549 and particularly LL 29, which is the cystic fibroblast cell model, suggests the potential use of these NPs for pulmonary delivery.

To investigate the potential anti-adhesive action of mBTL and mBTL-loaded CANPs, the adhesion ability of bacterial isolates in the presence of mBTL and mBTL-loaded NPs to human lung A549 cells was studied in the present study. As presented in the adhesion assays results, the mBTL alone reduced the adhesion of wild-type PA01, lasR, lasI, rhIR, and lasA in a dose-dependent pattern and inhibited the adhesion of double mutants to A549 cells. Upon the loading of mBTL into CANPs, the number of bacteria adherent to A549 cells was reduced in a pattern comparable with that of mBTL. Of note, the mBTL inhibited the adhesion of the ΔlasR ΔrhlR double mutant to A549. This mutant was reported to be less virulent owing to its lack of LasR and RhlR genes, the two principal transcriptional regulators of the *P. aeruginosa* QS system [36]. In this regard, it is well known that adhesion contributes to the attachment and invasion of *Pseudomonas aeruginosa* to epithelial cells. The data of our results show that mBTL-loaded CANPs reduced *P. aeruginosa* adhesion to A549 cells, suggesting their potential in the treatment and prophylaxis of bacterial infection.

Considering the promising observed improved in vitro anti-pneumococcal activity of mBTL loaded into CANPs, further in vivo research would provide a higher level of clinical relevance. Further investigation of the formulation on an animal infection model to support the potential use of mBTL-loaded CANPs for the prophylaxis and treatment of *P. aeruginosa* infection is recommended. 

## 5. Conclusions

The development of antimicrobial resistance among pathogenic bacteria, including *P. aeruginosa*, requires a new strategy, such as the use of non-antibiotics such as QSI, to combat the infection without inducing resistance. In this study, the prepared mBTL-CANPs showed higher antibacterial activity when compared with mBTL alone against tested bacterial isolates. The findings also show that mBTL-CANPs exhibited anti-virulence activity, including the reduction of biofilm formation and production of pyocyanin against wild-type *P. aeruginosa* and QS-deficient mutants. The formulated mBTL-CANPs at MIC_50_ were cytocompatible in the A549 and LL 29 cell lines and decreased the cytotoxic effects shown with mBTL alone on these cells. In a pattern comparable with mBTL alone, the NPs synthesized reduced the *P. aeruginosa* strains’ adhesion to A549, implying their potential anti-adhesive effect. Considering the mBTL-CANPs’ enhanced anti-pseudomonal effect, cytocompatibility, and anti-virulence suppressing action, as well as the advantage of alginate-based NPs as an antimicrobial delivery system, the formulated nanoparticles have significant potential for the prophylaxis and treatment of *P. aeruginosa* infection.

## Figures and Tables

**Table 1:** Bacterial strains used in this research.

Bacterial Strain	Abbreviation	Description	Reference
PA01	PA01	PA01 wild-type	[21]
PA1430	*ΔlasR*	Transcription regulator LasR	[21]
PA1432	*ΔlasI*	Autoinducer synthesis protein LasI	[21]
PA1871	*ΔlasA*	LasA protease precursor	[21]
PA3477	*ΔrhIR*	Transcriptional regulator RhIR	[21]
D6A	*ΔlasR ΔrhlR* double mutant	ΔlasR ΔrhlR knockouts double mutant	[21]

## Data Availability

The data presented in this study are available on request from the corresponding author.
